# Bijel Membranes with Tunable Porosity for pH‐responsive Microfiltration

**DOI:** 10.1002/smll.202504768

**Published:** 2025-07-16

**Authors:** Henrik Siegel, Martin F. Haase

**Affiliations:** ^1^ Van't Hoff Laboratory of Physical and Colloid Chemistry Debye Institute for Nanomaterials Science Department of Chemistry Utrecht University Utrecht 3584 CH The Netherlands

**Keywords:** bijels, composites, hydrogel, nanoparticles, separation membranes, wetting

## Abstract

Bicontinuous interfacially jammed emulsion gels (bijels) are nanoparticle‐stabilized composite materials with significant potential in membrane separations. This work introduces the fabrication of bijel sheets with symmetric pore structures and demonstrates their use as pH‐responsive microfiltration membranes. Unlike established hollow fiber‐bijel membranes, symmetric bijel sheets offer a more scalable and robust production route of separation membranes via roll‐to‐roll solvent transfer induced phase separation (R2R‐STrIPS). The role of substrate wetting by the liquid bijel precursor in determining membrane structure is analyzed. Pore sizes are tuned by varying the surfactant concentration in the precursor solution. In contrast to existing bijel membranes that rely on rigid polymer scaffolds ‐ resulting in brittleness and limited control over separation properties, stimuli‐responsive polymers are employed to create soft, adaptable bijels. This pH‐responsiveness enables dynamic control over membrane permeability, including feed‐stream‐driven compaction and tunable performance via polymer cross‐linking. These functional characteristics expand the potential applications of symmetric bijel sheets to drug delivery, controlled release, and soft robotics.

## Graphical abstract

This article reports the scalable production of symmetric bijel sheets with tunable, pH‐responsive pores for microfiltration. Unlike brittle, rigid bijel membranes, these soft, adaptable sheets enable dynamic control of permeability via pH changes. This robust roll‐to‐roll fabrication approach expands potential applications in separation membranes, drug delivery, controlled release systems, and soft robotics.

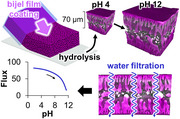

## 1 Introduction

Nanocomposite separation membranes are synthetic materials composed of nanoparticles and polymers with applications in water treatment,^[^ ^1^ ^]^ catalysis,^[^ ^2^ ^]^ and chemical production.^[^ ^3^ ^]^ Such membranes are made by incorporating inorganic nanoparticles (NPs) into the polymer to reinforce the polymer matrix and refine the membrane properties.^[^ ^4^, ^5^, ^6^ ^]^ NP additives can, for instance, increase the membrane fouling resistance, impart chemical stability, and provide adsorptive capabilities.^[^ ^7^, ^8^, ^9^ ^]^ The improved separation properties arise from interactions between the separation feed and the NPs located in the polymer scaffold and at the membrane surface. Therefore, fabricating nanocomposite membranes requires materials and synthesis methods that ensure dense and uniform NP distributions across the membrane.^[^ ^8^, ^10^ ^]^

In recent years, bicontinuous interfacially jammed emulsion gels (bijels) have emerged as a promising material to generate nanocomposite membranes with dense NP coatings.^[^ ^11^, ^12^, ^13^, ^14^ ^]^ Bijel membranes are made via solvent transfer induced phase separation (STrIPS).^[^ ^15^, ^16^ ^]^ During STrIPS, the phase separation of a mixture of oil, water, and solvent is arrested by surfactant‐modified NPs that form jammed layers at the oil/water interface.^[^ ^11^, ^15^ ^]^ The NP‐decorated bijel is solidified through polymerization of the oil. Various techniques have been employed to produce bijels via STrIPS, including fiber spinning,^[^ ^11^, ^12^, ^13^, ^14^, ^17^ ^]^ doctor‐blade casting,^[^ ^18^ ^]^ and roll‐to‐roll film coating (R2R‐STrIPS).^[^ ^19^, ^20^ ^]^ R2R‐STrIPS enables the continuous fabrication of flat‐sheet bijels at scalable dimensions, facilitating their use as a separation membrane.

Bijel formation via R2R‐STrIPS involves the coating of the precursor mixture as tens of micrometers thin liquid film onto a substrate in water. Previous studies have investigated the effect of precursor liquid composition and NP‐surfactant modification on the structure of bijels formed through STrIPS in a bulk liquid.^[^ ^11^, ^13^, ^19^, ^21^ ^]^ However, in R2R‐STrIPS, the phase separation of the precursor occurs at the surface of the substrate. Solid surfaces are known to affect the phase separation kinetics of fluid mixtures by the preferential wetting of the surface by one of the liquid components.^[^ ^22^, ^23^, ^24^ ^]^ Additionally, due to the low precursor viscosity, substrate wettability has been shown to cause macroscopic deformations of the bijel during R2R‐STrIPS.^[^ ^19^ ^]^ As the bijel structure determines the functional properties of the membrane, controlling the interplay of phase separation and substrate wetting is crucial for the synthesis of flat‐sheet bijels with well‐defined porosity.

In this article, we study how substrate wetting by the liquid bijel precursor governs the phase separation dynamics and structure formation of bijel membranes during R2R‐STrIPS. Wetting experiments show that on more hydrophobic substrates, the spreading of precursor mixture is facilitated during STrIPS, resulting in bijel membranes with asymmetric pore structures, which remain adhered to the R2R‐substrate. In contrast, substrate wetting by water leads to the formation of symmetric bijel membranes, which detach from the substrate as the phase separation can proceed toward both R2R‐water bulk and substrate. Confocal microscopy analysis shows that the bijel pore sizes can be controlled by increasing the surfactant concentration in the precursor, enabling the use of the substrate‐free bijel membranes for microfiltration. To regulate the permeability during filtration, we demonstrate that the bijel polymer matrix can be hydrolyzed, producing polyacrylic acid (PAA) hydrogels with pH‐sensitive water flux. Filtration tests show that for a given pH, the water flux of the bijel‐derived hydrogel depends on the PAA cross‐linker concentration. The ability to control the bijel structure through substrate wetting and to derive hydrogels for flux regulation promotes the formation of bijel membranes via R2R‐coating for applications in filtration, controlled release, and tissue engineering.

## 2 Results and Discussion

### 2.1 Bijel Film Synthesis

**Figure 1:**
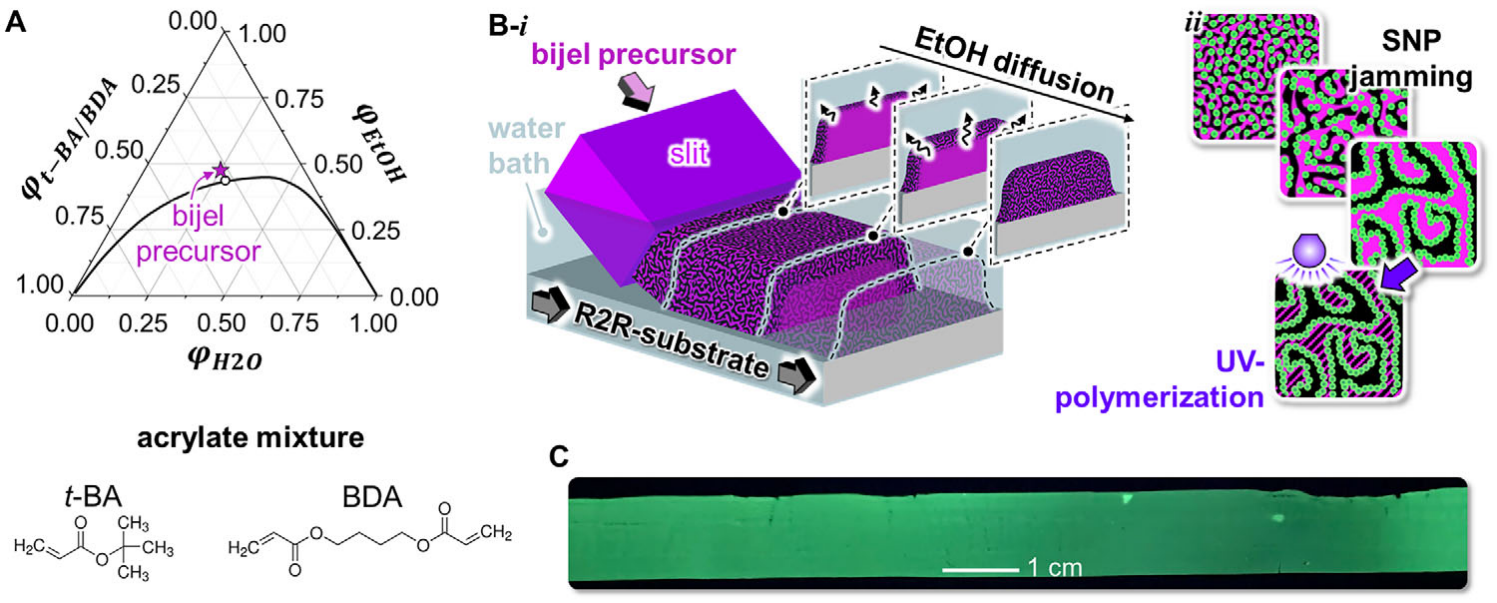
Bijel film fabrication via R2R‐STrIPS. A) Ternary liquid phase diagram of a mixture of *tertiary*‐butyl acrylate (*t‐*BA; 92 wt.%) and 1,4‐butanediol diacrylate (BDA; 8 wt.%), water, and ethanol (EtOH) plotted in liquid volume fractions *φ*. The critical point is given as a white dot. B‐i) Bijel film fabrication via roll‐to‐roll (R2R)‐STrIPS. ii) Sketches showing the silica nanoparticle (SNP) jamming of the *t‐*BA‐*co*‐BDA/water interface. Magenta color represents *t‐*BA‐*co*‐BDA, black color water, and green color SNPs. C) Fluorescent photograph of polymerized bijel film.

We synthesize bijel films via R2R‐STrIPS.^[^ ^19^ ^]^ To this end, we prepare the bijel precursor by mixing the liquids *tertiary*‐butyl acrylate (*t*‐BA), 1,4‐butanediol diacrylate (BDA), and water using ethanol as solvent at the composition specified in the ternary phase diagram in **Figure** **1**A (see Section S1, Supporting Information). In this liquid precursor mixture, we disperse 33 wt.% silica nanoparticles (Ludox TMA, 20 nm, acidified to pH 1.7; abbreviated as SNPs) together with cetyltrimethylammonium bromide (CTA^+^) as surfactant.

For R2R‐STrIPS, the precursor mixture is pumped through a slit onto polyethylene terephthalate (PET) substrate submerged in water (pH 1.7; Figure 1B‐i). Ethanol diffuses from the precursor mixture into the bulk water as the mixture exits the slit. The diffusion of ethanol triggers phase separation in the precursor mixture, generating a phase rich in *t*‐BA/BDA and an aqueous phase. As illustrated in Figure 1B‐ii, phase separation is halted by the jamming of CTA^+^‐modified SNPs at the interface between *t‐*BA/BDA and water. We solidify the resulting bijel film by polymerizing the *t‐*BA/BDA monomers with high‐intensity UV‐light. The cross‐linking density can be tuned via the *t‐*BA/BDA ratio. Figure 1C shows a photograph of the polymerized bijel film with green fluorescence of Coumarin 6 dye embedded in poly(*t‐*BA‐*co*‐BDA) under UV‐light.

After R2R‐STrIPS we collect the polymerized bijel film along with the PET substrate. To enable applications as filtration material, the bijel must be detached from the PET because the substrate blocks the flow of liquid. However, we cannot remove the PET because the bijel film remains adhered to it. Interestingly, when R2R‐STrIPS is repeated with cellulose as the substrate material, the resulting bijel film can easily be stripped off. What is the reason for this difference in bijel adhesion?

### 2.2 Bijel Adhesion and Substrate Wetting

We speculate that precursor wetting on PET causes the poly(*t‐*BA‐*co*‐BDA)‐bijel to adhere to the substrate, while precursor de‐wetting on cellulose facilitates detachment of the bijel film.^[^ ^19^ ^]^ PET is a hydrophobic plastic, whereas cellulose is a hydrophilic polymer. To gain a more detailed understanding of the bijel adhesion and precursor wetting, we perform the R2R‐coating on a third substrate material with intermediate polarity. We select cellulose acetate for this because the acetyl groups potentially alter the wettability by the bijel precursor compared to cellulose. In the following, we investigate the bijel film adhesion on the different substrates and show that the substrate material has a major impact on the phase separation process and the bijel structure.

**Figure 2:**
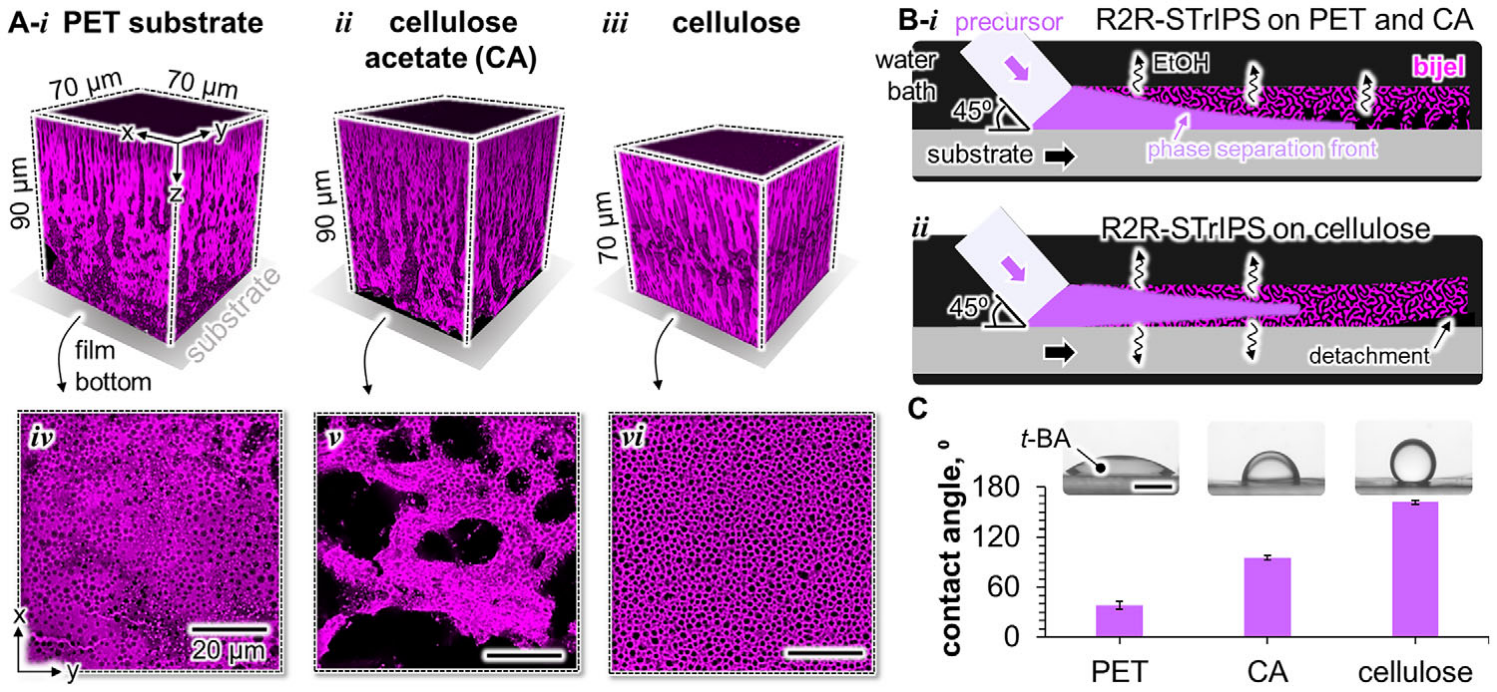
Bijel adhesion and substrate wetting. A) 3D confocal laser scanning microscopy (CLSM) images of bijel films made on the R2R‐substrates i) PET, ii) cellulose acetate (abbreviated as CA), and iii) cellulose. iv–vi) show bijel structures formed at the substrate interface. Poly (*t‐*BA‐*co*‐BDA) is colored in magenta and water in black. B) Schematic representation of the bijel film formation on i) PET and CA, and ii) cellulose. C) Contact angles of pure *t‐*BA on the different substrate materials in water (pH 1.7) with photographs of sessile *t‐*BA droplets. Black scale bar is 1 mm. The error bars represent the standard deviation of the average contact angle from three independent measurements.

R2R‐STrIPS produces macroscopically uniform bijel films with straight edges on all substrates, but confocal laser scanning microscopy (CLSM) reveals striking differences in the bijel microstructures. We observe that the bijel detaches from cellulose already before UV‐polymerization, while it remains adhered to PET and cellulose acetate even after polymerization. The bijel also detaches from cellulose substrate with mechanically enhanced surface roughness (Figure S2, Supporting Information). To analyze the bijel film structures, we label the poly(*t‐*BA‐*co*‐BDA) domains with the fluorescence dye Nile red for CLSM imaging. **Figure** **2**A‐i shows a 3D‐CLSM reconstruction of the bijel film prepared on PET with poly(*t‐*BA‐*co*‐BDA) colored in magenta and black water pores. The bijel features small pores at the surface and larger pores stretching toward the bottom. A similar bulk structure with vertically elongated pores is observed for the bijel made on cellulose acetate (Figure 2A‐ii). However, the bijel formed on cellulose has a remarkably different structure, displaying small pores on both sides of the film (Figure 2A‐iii). Hence, asymmetric bijel membranes are formed on PET and cellulose acetate, whereas symmetric membranes are produced on cellulose.

To connect our observations on adhesion behavior and bijel structure to the properties of the substrate, we inspect the arrangement of poly(*t‐*BA‐*co*‐BDA) and water that has formed at the interface between bijel and PET, cellulose acetate, and cellulose during R2R‐STrIPS. Figure 2A‐iv shows that the bottom plane of the bijel fabricated on PET consists of a poly(*t‐*BA‐*co*‐BDA)‐rich layer with round water inclusions. In comparison, the structure arrested on cellulose acetate contains much larger water pores and a perforated poly(*t‐*BA‐*co*‐BDA) matrix (Figure 2A‐v). At the bijel‐cellulose interface depicted in Figure 2A‐vi, we observe small water pores separated by a thin scaffold of poly(*t‐*BA‐*co*‐BDA). How does the substrate influence the formation of these different bijel structures?

The CLSM images in Figure 2A‐iv–vi indicate that substrate wetting influences the arrangement of oil and water at the substrate/precursor mixture interface. During STrIPS, the *t*‐BA/BDA and water‐rich phases can distribute in the liquid precursor film before the demixing is arrested. On PET and cellulose acetate, the delayed diffusion of ethanol from deeper inside the film results in the formation of larger *t*‐BA/BDA and water domains at the bijel bottom, as illustrated in Figure 2B‐i.^[^ ^13^ ^]^ STrIPS generates primarily *t‐*BA/BDA domains on the hydrophobic PET substrate, and mixed water/(*t‐*BA/BDA) domains on cellulose acetate. On cellulose, the fine poly(*t*‐BA‐*co*‐BDA) matrix with discrete water pores suggests that the substrate triggers STrIPS at the cellulose/precursor mixture interface (Figure 2B‐ii). The hydrophilic nature of cellulose can facilitate the uptake of water from the R2R‐water bulk into the substrate and therefore, promote the diffusion of ethanol from the bijel precursor during STrIPS. To relate the different distributions of oil and water to the substrate wetting, we measure the contact angles of *t*‐BA on the various substrates as discussed next.

The contact angle measurements of *t*‐BA on the substrates in water confirm that the PET surface can be wetted by the acrylate monomers. The photographs of the sessile *t*‐BA droplets in Figure 2C demonstrate that *t*‐BA spreads on PET, as reflected in a contact angle of 39 ± 5°. *t*‐BA partially wets the surface of cellulose acetate as indicated by a contact angle of 95 ± 3°, but de‐wets on cellulose, resulting in a contact angle of 162 ± 2°. Given the preferential wetting on PET, *t‐*BA/BDA can potentially accumulate at the substrate interface during STrIPS and form a wetting layer before the SNPs arrest the phase separation. Near the bijel‐PET interface, small pores form via nucleation of water droplets (Figure 2A‐iv).^[^ ^25^ ^]^ On cellulose acetate, the pores can coarsen at the substrate interface as wetting by *t*‐BA is less favourable. In contrast, on cellulose, ethanol diffusion via the substrate interface promotes early stabilization of the phase separation, resulting in smaller pores at the bijel bottom.

We conclude that preferential substrate wetting by precursor mixture and R2R‐water bulk governs the phase separation dynamics. On PET, the delayed stabilization of the phase separation near the substrate and the wettability by *t*‐BA likely facilitate the formation of a poly(*t‐*BA‐*co*‐BDA)‐rich layer at the substrate interface, which can explain the adhesion of the bijel film. For cellulose acetate, the prolonged precursor phase separation and partial wettability by *t*‐BA can cause the accumulation of water above the substrate. The bijel film still adheres to cellulose acetate presumably due to the coarsened poly(*t‐*BA‐*co*‐BDA) matrix stretching across the surface of the substrate. In contrast, the hydrophilicity of cellulose can promote substrate wetting by the R2R‐water bulk, causing precursor phase separation to occur via both sides of the bijel and facilitating *t*‐BA de‐wetting. As a consequence, the bijel film detaches from cellulose.

After polymerization, the bijel coated on cellulose can be removed from the substrate without damaging the film. In contrast, the bijel film breaks into fragments when removing the PET and cellulose acetate substrate. For applications as a filtration membrane, the relatively dense bijel pore structure may require high filtration pressures, which can break the bijel membrane. In the following section, we show that a more open‐porous bijel structure can be readily obtained by controlling the concentration of CTA^+^ surfactant in the precursor mixture. For these experiments, we prepare bijel films exclusively on cellulose because these films can be detached from the substrate and tested for filtration.

### 2.3 Effect of CTA^+^ Concentration on Bijel Structure

**Figure 3:**
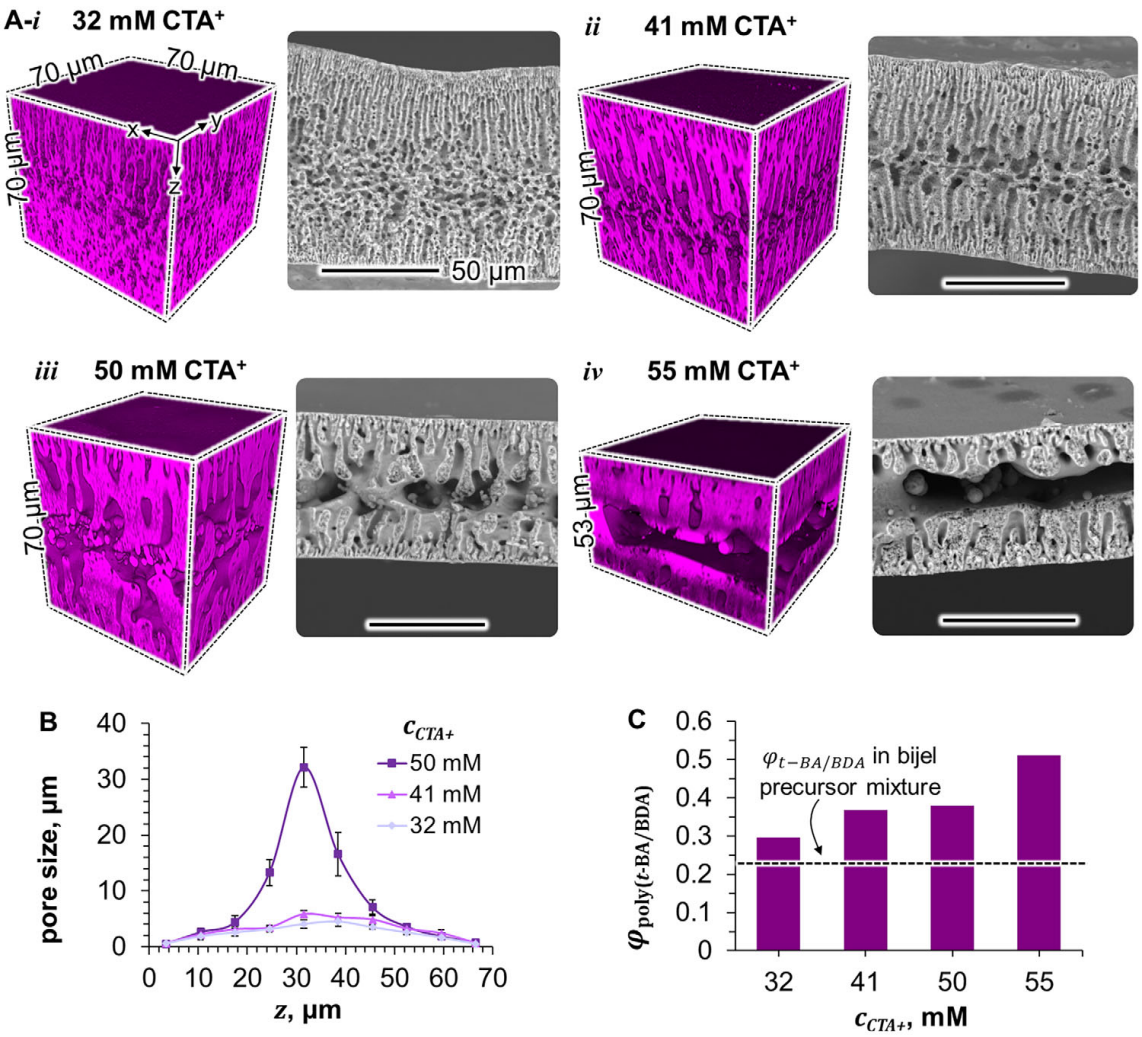
Effect of CTA^±^ concentration on bijel film structure. A) 3D‐CSLM and SEM images of bijel films prepared with different CTA^+^ concentrations (i–iv) in the precursor mixture. Black scale bars are 50 µm. B) Bijel pore size against film depth *z*. Error bars represent the standard deviation of the average pore size within the membrane interior of the bijels shown in (A). The lines are plotted as a guide for the eyes. C poly(*t‐*BA‐*co*‐BDA) volume fraction φ_*poly*(*t* − *BA*/*BDA*)_ measured for the bijel films in (A).

We fabricate bijel films from precursor mixtures containing 32–55 mm CTA^+^ and 33 wt.% SNPs at the liquid composition given in Figure 1A. CLSM and SEM analysis reveal that the CTA^+^ concentration has a pronounced effect on the bijel porosity. The 3D‐CLSM image of the bijel film made with 32 mm CTA^+^ in **Figure** **3**A‐i shows a dense network of poly(*t‐*BA‐*co*‐BDA) with interspersed water pores. Increasing the CTA^+^ concentration to 41 mm leads to the formation of more tubular water pores within the poly(*t‐*BA‐*co*‐BDA) matrix (Figure 3A‐ii). At 50 mm CTA^+^, large water pores merge into an interwoven arrangement with poly(*t‐*BA‐*co*‐BDA) featured in Figure 3A‐iii. In contrast, at 55 mm CTA^+^ thinner bijel films are formed consisting of a poly(*t‐*BA‐*co*‐BDA)‐rich shell and a virtually hollow interior (Figure 3A‐iv). CLSM images of film replicates are provided in Figure S3 (Supporting Information), and complementary SEM images are compiled in Figure S4 (Supporting Information).

Increasing the CTA^+^ concentration in the precursor mixture produces bijels with larger internal pores. At mid‐depth between the outer film surfaces, the water pore sizes increase from 4 ± 1 µm for the bijel made with 32 mm CTA^+^ to 32 ± 4 µm at 50 mm CTA^+^ (Figure 3B). This coarsening of the bijel pores can result from a delayed SNP jamming during phase separation, likely caused by a stronger hydrophobization of the SNPs at higher CTA^+^ concentrations^[^ ^13^, ^21^ ^]^ due to electrostatic and cooperative surfactant adsorption on the particle surface.^[^ ^26^, ^27^, ^28^ ^]^ More hydrophobic SNPs curve the interface toward the water‐rich phase,^[^ ^29^ ^]^ which is reflected by the round and oval water pores that have formed within the poly(*t‐*BA‐*co*‐BDA) scaffold across all CTA^+^ concentrations. Complementary contact angle measurements on SNP‐coated surfaces in water show that higher CTA^+^ concentrations facilitate particle wetting by *t‐*BA/BDA, supporting that increasing surfactant amounts render the SNPs more hydrophobic (Figure S7, Supporting Information).

However, in the center of the bijels made with 50–55 mm CTA^+^, the interface is also curved toward the oil, which suggests hydrophilic SNPs as compared to 32–41 mm CTA^+^. The change in SNP hydrophobicity can potentially result from the formation of adsorbed CTA^+^ bilayers at higher surfactant concentrations.^[^ ^27^ ^]^

Additionally, the CTA^+^ modification can promote the partitioning of SNPs into the *t*‐BA/BDA domains of the bijel. We infer the distribution of SNPs from measuring the poly(*t‐*BA‐*co*‐BDA) volume fraction φ_*poly*(*t* − *BA*/*BDA*)_ of the bijels over the entire thickness of the film, but excluding the outer surfaces. The poly(*t‐*BA‐*co*‐BDA) phase includes the *t*‐BA/BDA domains together with potentially residing SNPs. φ_*poly*(*t* − *BA*/*BDA*)_ increases from 0.3 for the bijel film made with 32 mm CTA^+^ to 0.5 at 55 mm CTA^+^ as plotted in Figure 3C. Compared to a *t*‐BA/BDA liquid volume fraction of 0.23 and a SNP volume fraction of 0.25 in the bijel precursor, the increase in φ_*poly*(*t* − *BA*/*BDA*)_ suggests that more surfactant facilitates the distribution of SNPs into the *t*‐BA/BDA‐rich phase.

The accumulation of SNPs in the *t*‐BA/BDA‐rich phase can potentially explain the increasing pore sizes at higher CTA^+^ concentrations. With an increasing amount of CTA^+^ the partitioning of SNPs into the *t*‐BA/BDA‐rich phase can reduce their availability to arrest the phase separation.^[^ ^13^ ^]^ This depletion of SNPs combined with the delayed ethanol diffusion in the center of the bijel can lead to the coarsening of *t*‐BA/BDA‐ and water‐rich domains at higher CTA^+^ concentrations. In addition to varying the surfactant concentration, reducing the SNP concentration in the precursor can also yield larger bijel pores (Figure S6, Supporting Information) as fewer particles are available to stabilize the phase separation.^[^ ^12^, ^13^, ^20^ ^]^ Notably, at 50 mm CTA^+^, bijel films are formed with water pores extending from the top to the bottom side, surrounded by a bulky scaffold of poly(*t‐*BA‐*co*‐BDA). In the next section, we show that this bijel structure enables microfiltration and demonstrate that the poly(*t‐*BA‐*co*‐BDA) matrix can be converted into a hydrogel to create membranes with pH‐responsive water flux.

### 2.4 Microfiltration and pH‐Responsive Water Flux

**Figure 4:**
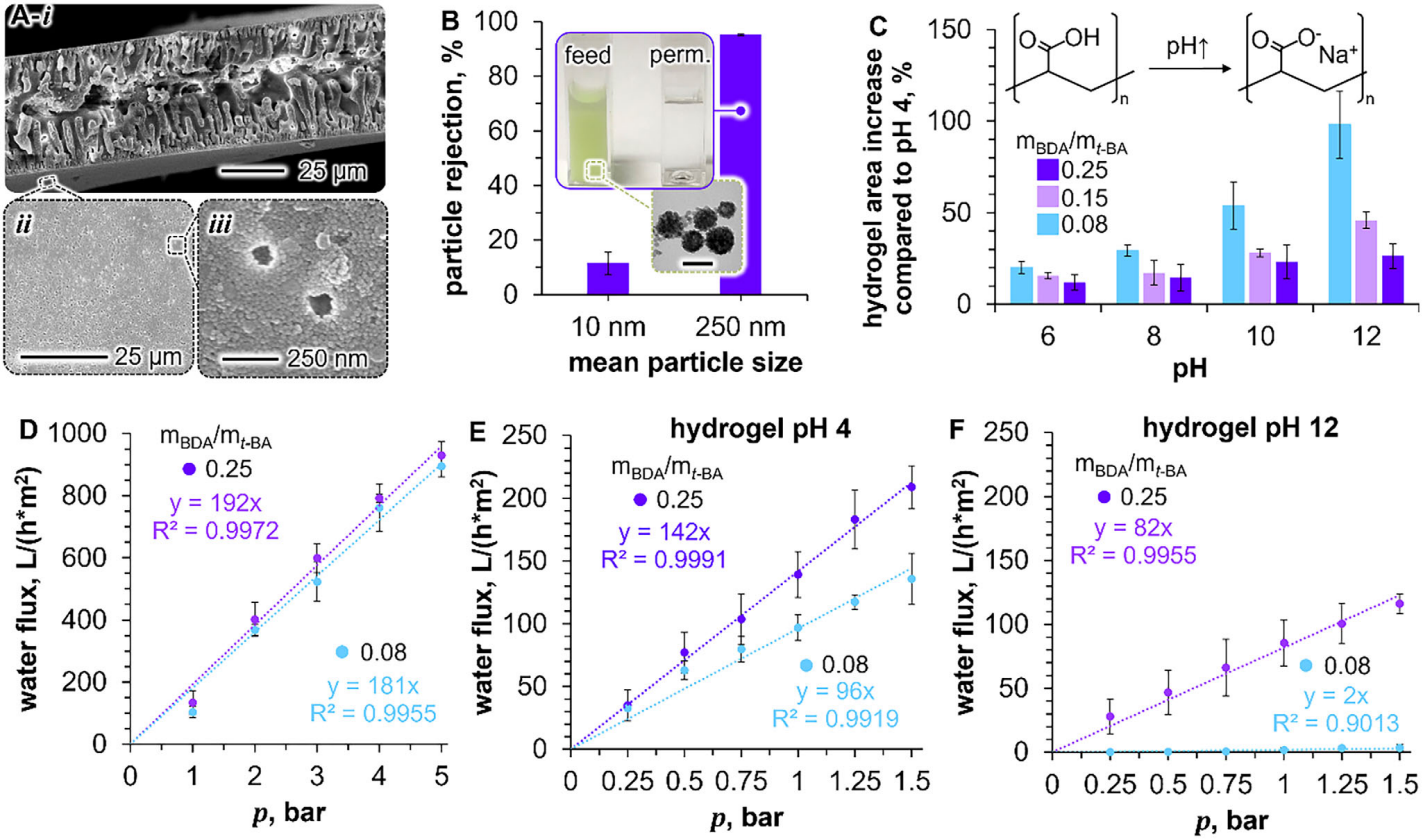
Microfiltration and pH‐responsive flux control. A‐i) SEM image of the cross‐section of the poly(*t*‐BA‐*co*‐BDA)‐bijel film prepared with 50 mm CTA^+^. ii, iii) Magnified insets of the film surface. B) Particle rejection during bijel membrane filtration. Photograph of SNP/polymer composite particle feed and permeate with transmission electron microscopy image of the composite particles. Black scale bar is 200 nm. C) Percentage area increase of polyacrylic acid hydrogels upon swelling at various water pH and cross‐linker ratios m_BDA_/m*_t_*_‐BA_. Error bars represent the standard deviation of the swelling measured from three individual samples for each pH and m_BDA_/m*_t_*_‐BA_ concentration tested. D) Water flux of poly(*t*‐BA‐*co*‐BDA)‐bijel membrane at different pressures and ratios m_BDA_/m*_t_*_‐BA_ (pH 4). Error bars give the standard deviation of the flux from three membrane samples. E) Water flux of hydrogels at different m_BDA_/m*_t_*_‐BA_ and pH 4 and F) pH 12 with error bars giving the standard deviation of the flux measured from three individual samples.

The scanning electron microscopy (SEM) image in **Figure** **4**A‐i shows the macroporous cross‐section of the polymerized bijel film fabricated with 50 mm CTA^+^. The permeable pore structure and the SNP‐encrusted film surface featured in Figure 4A‐ii,iii suggest microfiltration properties (Figure S11, Supporting Information). We test the bijel filtration performance by the separation of nanoparticles from aqueous suspensions (pH 4). To this end, we prepare two feed dispersions, one with SNPs of 10 nm mean size dispersed in water and another containing SNP/polymer composite particles with a mean size of 250 nm (see Section S11, Supporting Information). Prior to filtration, we wash the bijel membrane in an acidic water/ethanol solution to remove residual CTA^+^ to prevent SNP aggregation during filtration.^[^ ^13^ ^]^ The SNP dispersions are flown separately across the bijel in a dead‐end filtration cell at a pressure of 4 bar.

We present the particle rejection rates in Figure 4B. While only 12 ± 4% of the 10 nm SNPs are removed by the bijel, the membrane rejects 95 ± 1% of the SNP/polymer particles. The particle separation becomes visually evident when comparing the transparent permeate sample to the turbid SNP/polymer particle feed in Figure 4B, with the yellow/green color stemming from Coumarin 6 dye incorporated in the polymer. We conclude that most of the 10 nm SNPs pass through the poly(*t*‐BA‐*co*‐BDA)‐bijel membrane, while the SNP/polymer particles are likely sieved out by the bijel surface covered by a dense layer of SNPs. Although the rejected particles accumulate on the bijel surface and potentially clog the pores during dead‐end filtration, the dense SNP crust on both sides of the bijel membrane supports microfiltration capabilities. For bijel fiber membranes, the nanoparticle crust has been shown to control the separation selectivity during micro‐ and ultrafiltration, with the nanoparticle‐filled pores withstanding filtration pressures up to 4 bar.^[^ ^12^, ^13^ ^]^

After microfiltration, the porous morphology of the poly(*t*‐BA‐*co*‐BDA)‐bijel remains intact as indicated by the same membrane structure before and after filtration (Figure S12, Supporting Information). However, the rigid polyacrylate backbone also makes the membrane stiff and brittle. Intriguingly, we can transform the bijel into a flexible hydrogel through acid hydrolysis, converting poly(*t*‐BA) into polyacrylic acid (PAA) but maintaining the pore structure (Figure S8, Supporting Information).^[^ ^12^, ^17^ ^]^ The resulting PAA hydrogel is cross‐linked by butanediol diacrylate (BDA), which has been included in the bijel precursor mixture. Hydrogels are promising membrane materials due to their cross‐linked polymer networks, which can absorb water and modulate the membrane porosity.^[^ ^30^, ^31^, ^32^, ^33^ ^]^ In the following, we show that cross‐linking and water pH regulate the flux during filtration with bijel‐derived PAA hydrogels.

The pH controls the swelling of PAA hydrogels in water. Increasing the pH above the pKa of PAA (≈4.5) facilitates the deprotonation of the acrylic acid groups and triggers the uptake of water into the hydrogel.^[^ ^34^, ^35^ ^]^ The extent of swelling is controlled by the cross‐linking of the PAA network, which depends on the amount of cross‐linking agent incorporated into the hydrogel.^[^ ^35^, ^36^ ^]^ Based on these considerations, we test the effect of pH and BDA cross‐linker concentration on the swelling and water flow of bijel‐derived PAA hydrogels. The hydrogels are made from poly(*t*‐BA‐*co*‐BDA)‐bijel films containing varying amounts of BDA in the precursor mixture (given as weight fraction of the *t*‐BA solution m_BDA_/m*_t_*_‐BA_ = 0.08, 0.15, 0.25). After hydrolysis, the hydrogels are equilibrated in water adjusted to pH 4–12. We characterize the swelling by measuring the area expansion of the hydrogels as discussed next.

Figure 4C shows that at different pH, the m_BDA_/m*_t_*_‐BA_ ratio determines the extent of hydrogel swelling. When the pH increases from 4 to 12, the hydrogels made with m_BDA_/m*_t_*_‐BA_ = 0.08 swell to approximately twice their original area. In comparison, the hydrogels containing m_BDA_/m*_t_*_‐BA_ = 0.15 and 0.25 swell less, as indicated by a smaller area increase across the different pH values. While alkaline pH values facilitate the uptake of water into the hydrogel, higher ratios m_BDA_/m*_t_*_‐BA_ restrict this swelling.

We use the poly(*t*‐BA‐*co*‐BDA) membranes and hydrogels to measure their water flux at variable pressures. To avoid mechanical rupture during filtration, the membranes and hydrogels are placed on Nylon sheets during all measurements. Control experiments in Figure S13 (Supporting Information) show that the permeability of the Nylon support significantly exceeds the measured values here, indicating that the water flux is not obstructed by the support.

Figure 4D plots the water flux of the poly(*t*‐BA‐*co*‐BDA)‐bijel membranes containing the same m_BDA_/m*_t_*_‐BA_ ratios as the hydrogels. The slope of the linear fit of measured flux against pressure gives the membrane permeability. For the poly(*t*‐BA‐*co*‐BDA)‐bijels, the water permeability amounts to 180–190 L/(h*m^2^*bar) irrespective of m_BDA_/m*_t_*_‐BA_. In contrast, Figure 4E shows that the hydrogel permeability reaches 142 ± 17 L/(h*m^2^*bar) at m_BDA_/m*_t_*_‐BA_ = 0.25 and pH 4, and 96 ± 3 L/(h*m^2^*bar) at m_BDA_/m*_t_*_‐BA_ = 0.08. At pH 12 and m_BDA_/m*_t_*_‐BA_ = 0.25, the hydrogel permeability decreases to 82 ± 12 L/(h*m^2^*bar) (Figure 4F). For m_BDA_/m*_t_*_‐BA_ = 0.08, the permeability drops to 2 ± 1 L/(h*m^2^*bar) at pH 12. Thus, increasing the pH and lowering the cross‐linker concentration to m_BDA_/m*_t_*_‐BA_ = 0.08 allows for almost complete interruption of the water flow during hydrogel filtration.

Our measurements suggest that the rigid poly(*t*‐BA‐*co*‐BDA) membranes enable higher water flux than the PAA hydrogels during pressure filtration. The flux decrease of the hydrogels may result from the compression of the membrane at lower ratios m_BDA_/m*_t_*_‐BA_.^[^ ^30^, ^37^, ^38^, ^39^ ^]^ While the hydrogel surface pores are open before and after filtration (Figure S14, Supporting Information), more research is needed to detail how the pressure drop across the hydrogel affects membrane structure and fluid flow during pressure‐driven filtration. Previous work has shown that the elastic modulus of bijel‐templated PAA fibers ranges from 27 to 1700 kPa for cross‐linker concentrations increasing from 0.01 to 0.11, indicating that the hydrogel membranes feature low mechanical strength.^[^ ^17^ ^]^ The limited stability of the hydrogels is supported by the observation that some membranes break at pH 12 and m_BDA_/m*_t_*_‐BA_ = 0.08 when exposed to water pressures ≥ 1 bar for ≈30 min. Moreover, the hydrogels deform during repeated pH cycling (Figure S9, Supporting Information), restricting the application of the presented hydrogel membranes to single‐use applications. Based on our experiments, we conclude that adjusting the cross‐linker concentration imparts control over the water flux during pH‐sensitive filtration with bijel‐derived PAA hydrogels.

## 3 Conclusion

In this study, we investigate the effect of substrate wetting on the structure and phase separation of bicontinuous interfacially jammed emulsion gels (bijels) to produce microfiltration membranes with pH‐responsive permeability. Bijels are synthesized by coating the liquid precursor as a thin film onto polymer substrates using roll‐to‐roll solvent transfer induced phase separation (R2R‐STrIPS).^[^ ^19^ ^]^ Confocal microscopy analysis and contact angle measurements show that preferential substrate wetting by the bijel precursor results in membranes with asymmetric pore structures. In contrast, preferential wetting by the R2R‐water bulk produces symmetric bijel membranes that function as free‐standing microfiltration membranes with pore sizes controlled by the surfactant concentration in the precursor. We demonstrate that hydrolysis converts the bijel membrane into cross‐linked polyacrylic acid (PAA) hydrogels with pH‐responsive water permeability. The water flux of the hydrogels is controlled by the PAA cross‐linker concentration and decreases at alkaline solution pH. Modulating the bijel structure through substrate wetting together with the ability to manipulate water flux promotes the R2R fabrication of bijel membranes and bijel‐derived hydrogels for flow regulation,^[^ ^40^ ^]^ controlled release,^[^ ^41^, ^42^ ^]^ tissue engineering,^[^ ^43^, ^44^, ^45^ ^]^ or functional fabrics.^[^ ^46^ ^]^

## 4 Experimental Section

### Bijel Precursor Preparation

The bijel precursor was a homogeneous mixture composed of *tertiary*‐butyl acrylate (*t*‐BA; Alfa Aesa), water (MilliQ purification systems), ethanol (EtOH; Merck), Ludox TMA nanoparticles, and cetyltrimethylammonium bromide (CTA^+^). The *t*‐BA liquid contains 1 wt.% of the photo‐initiator 2‐hydroxy‐2‐methylpropiophenone (Sigma–Aldrich), the dye Nile red (Sigma–Aldrich), and various weight fractions of 1,4‐butanediol diacrylate (BDA; 8, 15, 25 wt.%; Sigma–Aldrich). First, 56.3, 71.9, 87.5, and 95.3 mg CTA^+^ were dissolved in 0.954 g of the *t*‐BA liquid to obtain precursor concentrations of 32, 41, 50, and 55 mm CTA^+^, respectively (see Table S2, Supporting Information).

Next, Ludox TMA nanoparticles (34 wt.% colloidal silica, 20 nm; Sigma–Aldrich) were incorporated into the precursor mixture. The nanoparticles were prepared as follows: 20 mL of the Ludox TMA dispersion was adjusted to pH 1.7 by the addition of 1 m HCl (Acros Organics) and dialyzed in EtOH overnight. After dialysis, the particle weight fraction (68.5 wt.%) was determined from measuring the mass before and after evaporation of liquid at 100 °C (IKA RCT heating plate). Separately, 40 mL Ludox TMA dispersion was concentrated by the evaporation of water (Rotary evaporator, Heidolph Instruments) at 60 °C and 140 mbar, yielding a particle concentration of 52.8 wt.%. Particle aggregates were removed by centrifuging the concentrate at 3270 rcf for 10 min (Allegra X‐12R; Beckman Coulter) followed by acidification to pH 1.7 using 1 m HCl.

EtOH of 1.177 g and 0.764 g of the nanoparticle dispersion dialyzed in EtOH were added to the precursor. The mixtures were placed on a heating plate for 15 min at 50 °C to facilitate dissolution of CTA^+^. Last, 2.226 g of the concentrated aqueous Ludox TMA dispersion and 0.078 g water (pH 1.7) were added, followed by 30 sec ultrasonication (Branson 1800) to disperse the nanoparticles in the bijel precursor.

### Bijel Film Fabrication

Bijel films were synthesized via R2R‐STrIPS.^[^ ^19^ ^]^ The bijel precursor mixture was pumped at 3.5 mL min^−1^ (AL‐300 syringe pump; World Precision Instruments) through a glass slit coated with 0.2 wt.% PDADMAC (250–350 kDa, 500 mm NaCl; Sigma–Aldrich) onto a substrate. The substrate was pulled at 5 cm s^−1^ through a tank filled with water of pH 1.7 and 5 vol% EtOH. As substrates, polyethylene terephthalate (PET; 100 µm thick, Reflectiv), cellulose acetate (Replicating tape, 22 µm thick, SPI Supplies), and cellulose (dialysis tubing; MWCO 14 kDa, Carl Roth) were used. The bijel films were polymerized by exposure to high‐intensity UV‐light (320–500 nm; OmniCure Series 1500).

### Bijel Film Structure Characterization

Confocal laser scanning microscopy (Stellaris 5; Leica Microsystems) was employed to analyze the bijel structure. The polymerized bijel films were immersed in dimethyl sulfoxide (Sigma–Aldrich), and the fluorescence of Nile red was excited with 561 nm laser light and detected at 600–750 nm. *z*‐stacks were acquired at a 0.4 µm focal step size. For scanning electron microscopy, the bijel films were dried and sputter‐coated with 6 nm Platinum (15 kV incident electron beam; Zeiss GeminiSEM 450).

To measure the bijel pore sizes, the CLSM images were binarized and thresholded using the software Fiji ImageJ (version 1.53k14). For each 8 µm slice along the *z*‐depth of the bijel film, water pore sizes were determined by measuring the width and length of the pores, calculating the arithemtic mean and averaging these mean values per slice. The outer bijel surfaces were excluded from analysis as the pores cannot be clearly identified in these regions. The poly(*t*‐BA‐*co*‐BDA) volume fraction was analyzed from pixel count analysis of the processed confocal micrographs (see Section S5, Supporting Information). Fiji ImageJ was employed to measure the number of pixels showing the poly(*t*‐BA‐*co*‐BDA) flourescence per slice. The pixel area per slice was converted into µm^2^ using the aspect ratio of the CLSM images (11.1 pxl µm^−1^) and multiplied by 0.4 µm to obtain the volume of poly(*t*‐BA‐*co*‐BDA). Dividing the poly(*t*‐BA‐*co*‐BDA) volume by the volume of the analyzed bijel film segment gives the volume fraction φ_*poly*(*t* − *BA*/*BDA*)_. The pore sizes were averaged per film depth, and the standard deviation was calculated.

### Contact Angle Measurements

*t*‐BA of 8 µL was dispensed via a syringe with attached needle (25 GA; Metcal) onto PET, cellulose acetate, and cellulose in water (pH 1.7) using a pendant drop tensiometer (Dataphysics OCA25 with SCA20 software) with Thorlabs CS165MU/M camera. The contact angle was measured by fitting the shape of the *t*‐BA droplet and averaging the contact angles obtained from measurements with three doplets.

### Microfiltration with Bijel Films

The polymerized bijel films (prepared with 50 mm CTA^+^; 25 wt.% BDA in the *t*‐BA solution) were washed for 2 h at 50 °C in a mixture of 50 vol% EtOH and 50 vol% 1 m HCl to remove residual CTA^+^. Film pieces of ≈8 mm×8 mm were cut out using a razor blade and assembled in a home‐built dead‐end filtration device (see Section S10, Supporting Information). Two aqueous dispersions were prepared as filtration feeds (pH 4): i) 5 wt.% Ludox TMA (34 wt.% stock; Grace, batch 1000374513) with an average particle size of 10 nm and ii) 2 wt.% dispersion of Ludox TMA‐covered polymerized composite particles with an average size of 250 nm. Particle sizes were determined via dynamic light scattering (Zetasizer Ultra, Malvern Pananaltiycal) and the SNP/polymer composite particles were imaged using transmission electron microscopy (Talos L120C, 120 kV acceleration voltage; Philipps). The SNP/polymer composite particle synthesis is detailed in Section S11 (Supporting Information).

The feed dispersions were flown separately across the bijel at a pressure of 4 bar, and permeate samples were collected from three different membranes. The bijel was supported on a Nylon sheet with 5 µm pore size (RS10539; Tisch Scientific). Control experiments (Section S11, Supporting Information) confirm that the Nylon support does not influence the particle rejection. Particle separation was determined from measuring the mass of particles remaining in the permeate after evaporation of water at 120 °C (IKA RCT heating plate). The particle rejection *R* is calculated from the particle weight fraction in feed *w_f_* and permeate *w_p_* as:

(1) $$R=\left(1-\frac{w_{p}}{w_{f}}\right)*100\%$$

### Bijel Hydrolysis and Swelling

Hydrolysis of the bijel films was carried out in a solution of 20 wt.% formic acid (Sigma–Aldrich) and 80 wt.% trifluoroacetic acid (Sigma–Aldrich) for 12 h. The so‐obtained hydrogels were washed three times each in EtOH and water, and then stored in water (pH 4). Hydrogel swelling was analyzed upon immersion in water adjusted to pH 6–12 by the addition of 0.1 m NaOH (Merck KGaA). The hydrogel area was measured from the width and length of three individual hydrogel samples after 15 min equilibration at the different pH (Mitutoyo caliper) and comparison to the initial hydrogel area at pH 4. This analysis was performed for three hydrogels prepared with BDA concentrations of m_BDA_/m*_t_*_‐BA_ = 0.08, 0.15, and 0.25, respectively.

### Hydrogel Permeability

The hydrogel and bijel membrane water permeability was measured using a home‐built filtration device. First, the hydrogel membranes were equilibrated to pH by immersion in water at pH 4–12 for 15 min. Then, the hydrogels were placed on a Nylon support (RS10539; Tisch Scientific) and assembled in the filtration device. Air was expelled from the device by flushing with water adjusted to the same pH as the hydrogels prior to flux measurements. The water flux was measured for aqueous feeds at pH 4 and 12 at pressures ranging from 0.25 to 1.5 bar. Additionally, the water flux was measured at a constant pressure of 1 bar for hydrogels at pH 6–10. All membranes were equilibrated for 5 min at each pressure, and permeate samples were collected for 2–5 min. The water flux was determined from the retrieved mass of water. By plotting the water flux against the filtration pressure, the water permeability follows from the slope of a linear fit. All flux measurements were formed as triplicates from which the average flux and the standard deviation were calculated.

### Declaration of Generative AI and AI‐Assisted Technologies in the Writing Process

During the preparation of this work, the authors used ChatGPT in order to improve the grammar of the main text. After using this tool/service, the authors reviewed and edited the content as needed and take full responsibility for the content of the publication.

## Conflict of Interest

The authors declare no conflict of interest.

## Supporting information



Supporting Information

## Data Availability

The data that support the findings of this study are available from the corresponding author upon reasonable request.
