# Supplementary material for: Bijel Membranes with Tunable Porosity for pH‐responsive Microfiltration
Source: Small. 2025 Jul 16;21(36):2504768. doi: 10.1002/smll.202504768 (PMC12423898; doi:10.1002/smll.202504768)
Supplement: Supplementary file 1 — Supporting Information [file SMLL-21-2504768-s001.pdf]

NANO · MICRO

small

Supporting Information

for *Small*, DOI 10.1002/smll.202504768

Bijel Membranes with Tunable Porosity for pH-responsive Microfiltration

Henrik Siegel and Martin F. Haase**

Supporting Information

Bijel membranes with tunable porosity for pH-responsive microfiltration

Henrik Siegel[1], Martin F. Haase[1*]*

[1] Van't Hoff Laboratory for Physical and Colloid Chemistry, Department of Chemistry, Debye Institute for Nanomaterials Science, Utrecht University, Utrecht, The Netherlands

* Correspondence: h.siegel@uu.nl, m.f.haase@uu.nl

Table of contents

S1. Measurement of the ternary phase diagram S2
S2. Bijel precursor preparation S3
S3. Bijel membrane preparation on cellulose with rough surface S4
S4. Bijel membrane replicates and pore size distribution S5
S5. Measurement of the poly(*t*-BA) volume fraction S5
S6. Effect of nanoparticle concentration on bijel film structure S6
S7. Silica nanoparticle hydrophobization by CTA^+ S6
S8. Hydrogel swelling S7
S9. Microfiltration and water permeability measurements S8
S10. Hydrogel surface porosity before and after filtration S10
S11. Characterization of filtration feed dispersions S11

S1. Measurement of the ternary phase diagram

The binodal curve for the ternary liquid system composed of *tertiary*-butyl acrylate (*t*-BA), water, and ethanol (EtOH) is determined *via* turbidimetry measurements. Water is gradually added to a mixture of *t*-BA and EtOH with known initial liquid weight fractions, using an analytical balance (Mettler AT250 Analytical balance; Mettler Toledo) until the mixture becomes cloudy. The liquid weight fractions at the transition from a transparent to a cloudy mixture are converted into liquid volume fractions based on the densities of *t*-BA (0.88 g/mL), water (0.99 g/mL) and EtOH (0.79 g/mL). **Figure S1A** shows the ternary phase diagram for the pure liquids *t*-BA/water/EtOH.

The location of the critical point is concluded from measuring the volume ratio of phase separated *t*-BA/water/EtOH mixtures along the binodal curve. Following the lever rule, at the critical point a phase separated mixture has equal volumes of oil-rich and water-rich fractions. We prepare ternary liquid mixtures just below the binodal curve at compositions indicated by stars in **Figure S1A**. These mixtures separate into a *t*-BA-rich phase on top and a water-rich phase on the bottom as shown in **Figure S1B**. To distinguish the two phases, we label the *t*-BA-rich phase in dark red by adding the dye Nile red. For liquid mixture 3, the phase separation yields equal volumes of *t*-BA-rich and water-rich phase. The critical point is φ(*t*-BA) = 0.27, φ(EtOH) = 0.45 and φ(water) = 0.28.

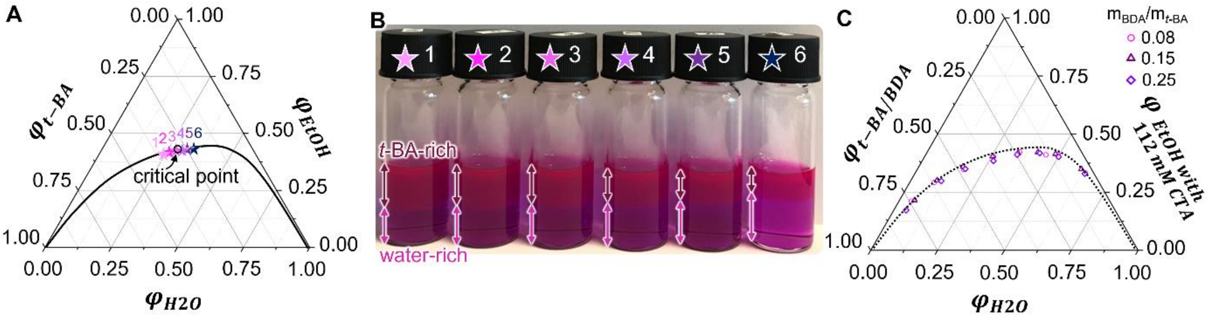

Figure S1. A Ternary liquid phase diagram for *tertiary*-butyl acrylate (*t*-BA), water, and ethanol (EtOH) given in liquid volume fractions φ. The stars numbered 1-6 represent the ternary liquid compositions used for the determination of the critical point. **B** Photographs of phase separated *t*-BA/water/EtOH mixtures at compositions denoted in A. **C** Ternary liquid phase diagram for *t*-BA, water, and EtOH with different weight fractions of 1,4-butanediol diacrylate (BDA) in *t*-BA (referred as $m_{BDA}/m_{t\text{-}BA}$) and 112 mM CTA^+ in EtOH. The black dashed line gives the binodal curve for the pure liquids.

To probe the effect of 1,4-butanediol diacrylate (BDA) on the miscibility of *t*-BA/water/EtOH, we measure the binodal curves for different BDA weight fractions incorporated in *t*-BA. We also dissolve 112 mM CTA^+ in EtOH to include the effect of CTA^+ on the liquid mixing behavior. The concentration of 112 mM CTA^+ in the EtOH volume fraction corresponds to a concentration of 50 mM in the precursor mixture. From turbidimetry measurements we obtain the liquid weight fractions for the binodal curve which are translated into volume fractions using the following densities: 0.90 g/mL for $m_{BDA}/m_{t\text{-}BA}$ = 0.08, 0.91 g/mL for $m_{BDA}/m_{t\text{-}BA}$ = 0.15, 0.92 g/mL for $m_{BDA}/m_{t\text{-}BA}$ = 0.25, and 0.80 g/mL for EtOH containing 112 mM CTA^+. BDA and CTA^+ have no significant effect on the binodal curve compared to the pure liquids (**Figure S1C**). The raw data for all ternary phase diagrams is provided in **Table S1**.

Table S1. Measured liquid volume fractions φ for the binodal curve of *t*-BA/water/EtOH.

φ(*t*-BA)	φ(EtOH)	φ(water)
0.9391	0.0537	0.0072
0.8773	0.1038	0.0189
0.7303	0.2180	0.0516
0.6256	0.2956	0.0788
0.4454	0.3863	0.1682
0.3803	0.4113	0.2085
0.2610	0.4438	0.2952
0.1767	0.4589	0.3643
0.0981	0.4465	0.4555
0.0475	0.4165	0.5360
0.0248	0.3498	0.6254
**φ(*t*-BA incl. 8 wt% BDA)**	**φ(EtOH incl. 112 mM CTA^+)**	**φ(water)**
0.7420	0.2114	0.0466
0.6164	0.2918	0.0918
0.4786	0.3485	0.1729
0.3387	0.4032	0.2581
0.2310	0.4229	0.3462
0.1393	0.4103	0.4505
0.0928	0.4118	0.4954
0.0298	0.3326	0.6376
**φ(*t*-BA incl. 15 wt% BDA)**	**φ(EtOH incl. 112 mM CTA^+)**	**φ(water)**
0.7279	0.2155	0.0565
0.6012	0.3009	0.0979
0.4743	0.3604	0.1653
0.3420	0.4043	0.2537
0.2400	0.4184	0.3416
0.1634	0.4222	0.4144
0.1014	0.4149	0.4837
0.0370	0.3493	0.6137
**φ(*t*-BA incl. 25 wt% BDA)**	**φ(EtOH incl. 112 mM CTA^+)**	**φ(water)**
0.7812	0.1723	0.0465
0.5866	0.2979	0.1155
0.4708	0.3501	0.1791
0.3484	0.3848	0.2668
0.2465	0.4116	0.3419
0.1584	0.4184	0.4232
0.0955	0.4023	0.5022
0.0340	0.3292	0.6368

S2. Bijel precursor preparation

Table S2 details the sequence of the bijel precursor preparation and reports the mass of all precursor components. The volume fraction of the pure liquids (excluding the volume of the silica nanoparticles; abbreviated as SNPs) is φ(*t*-BA) = 0.27, φ(EtOH) = 0.45 and φ(water) = 0.28.

Table S2. Bijel precursor preparation.

**Sample component**	**Precursor 32 mM CTA^+**	**Precursor 41 mM CTA^+**	**Precursor 50 mM CTA^+**	**Precursor 55 mM CTA^+**
CTA^+	56.3 mg	71.9 mg	87.5 mg	95.3 mg
t-BA (incl. 1 wt% HMPP, 8 wt% BDA and Nile red)	0.954 g	0.954 g	0.954 g	0.954 g
EtOH	1.177 g	1.177 g	1.177 g	1.177 g
68.5 wt% SNP dispersion (pH 1.7) dialyzed in EtOH	0.764 g	0.764 g	0.764 g	0.764 g
Mixing and heating at 50 °C				
52.8 wt% SNP dispersion (pH 1.7)	2.226 g	2.226 g	2.226 g	2.226 g
Water (pH 1.7)	0.078 g	0.078 g	0.078 g	0.078 g
Mixing and ultrasonication				

S3. Bijel membrane preparation on cellulose with rough surface

To test if surface roughness influences the adhesion of bijel films on cellulose substrate, we synthesized the bijel membrane on cellulose with mechanically enhanced surface roughness. The roughened cellulose is prepared by sanding with P80-grade sandpaper (Norton A275op; abrasive particle diameter of 190 – 265 µm according to FEPA standard). Sanding results in a visibly dull cellulose surface as shown in **Figure S2B-*i***. The roughened substrate is cleaned using pressurized air. On both smooth and rough cellulose, we cast the bijel precursor mixture containing 41 mM $C16TA^+$. In both cases, the bijel film detaches from the substrate prior to UV-polymerization. CLSM analysis confirms the formation of a symmetric pore structure on both substrates (**Figures S2A-*ii*** and **B-*ii***). The bijel film collected from the rough cellulose exhibits a curved bottom surface, which potentially results from the substrate texture. The cross-sectional insets of the bijel membrane structure in **Figures S2A-*iii*** and **B-*iii*** show a similar bijel microstructure at the interface to untreated and rough cellulose.

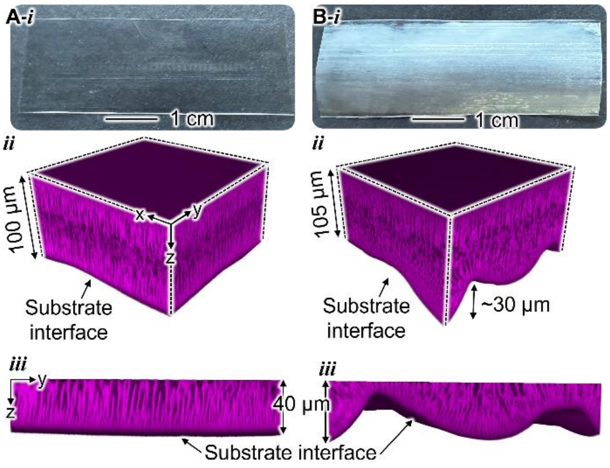

Figure S2. Bijel film synthesis on cellulose with **A-*i*** pristine and **B-*i*** sanded surface. **A-*ii*** and **B-*ii*** show 3D-CLSM images of the bijel films prepared on both substrates. **A-*iii*** and **B-*iii*** give cross-sectional projections of the bijel film directly above the substrate interface. Poly(*t*-BA-*co*-BDA) is colored in magenta and water in black.

S4. Bijel membrane replicates and pore size distribution

Figure S3 shows 3D-CLSM images of bijels prepared with the same precursor composition as the films presented in Figure 3A in the manuscript. The pore size distributions for the respective bijel replicates are plotted in **Figure S3A-*ii*** and **B-*ii***, featuring similar pore sizes and pore size profiles across the film depth.

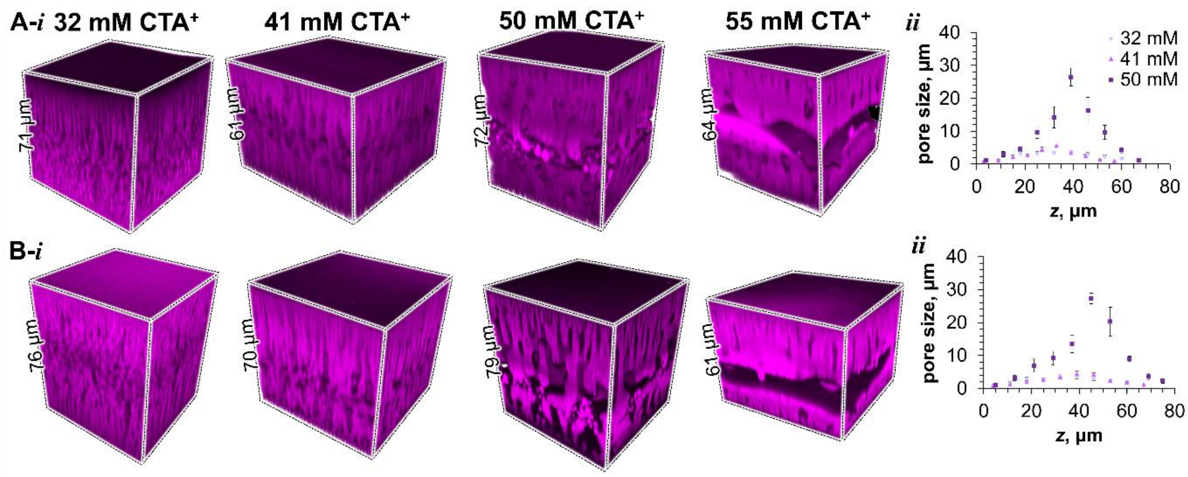

Figure S3. Bijel membrane replicates **A** and **B** prepared from precursor mixtures of different CTA^+ concentrations with their pore size distributions shown in **A-*ii*** and **B-*ii***.

Figure S4 shows representative scanning electron microscopy (SEM) images of the bijel films.

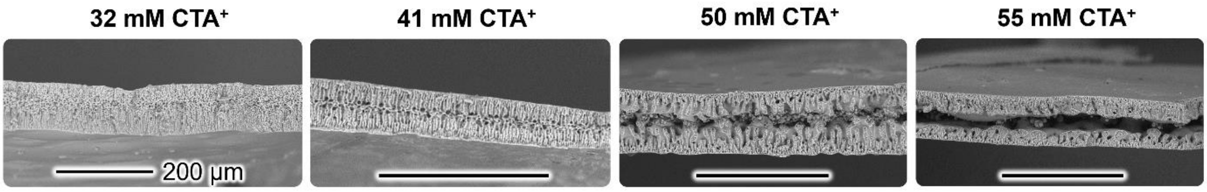

Figure S4. SEM images of the bijel films formed at different CTA^+ concentrations. Black scale bars are 200 µm.

S5. Measurement of the poly(*t*-BA) volume fraction

We calculate the poly(*t*-BA) volume fraction of the bijel from image analysis using the software Fiji ImageJ (version 1.53k14). The CLSM images capturing the Nile red fluorescence of the poly(*t*-BA) domains are processed according to **Figure S5A**. First, the image brightness and contrast is enhanced to distinguish poly(*t*-BA) from the background (**Figure S5A-*ii***). After running a bandpass filter, we set a gray scale threshold to accurately outline the poly(*t*-BA) domains and binarize the image (**Figure S5A-*iii***). The poly(*t*-BA) phase appears as white pixel while the water pores are black. With the known aspect ratio of each image (11.1 pxl/µm) the white pixel area is measured for each slice of the *z*-stack. The top and bottom surfaces of the bijel are excluded from analysis as the poly(*t*-BA) phase cannot be properly distinguished from the water pores. The resulting values for the white area are multiplied by the *z*-step size of 0.4 µm to calculate the volume of poly(*t*-BA) per slice (**Figure S5B**). The poly(*t*-BA) volume fraction $\varphi_{poly(t-BA)}$ is obtained by dividing the poly(*t*-BA) volume over the bijel film volume.

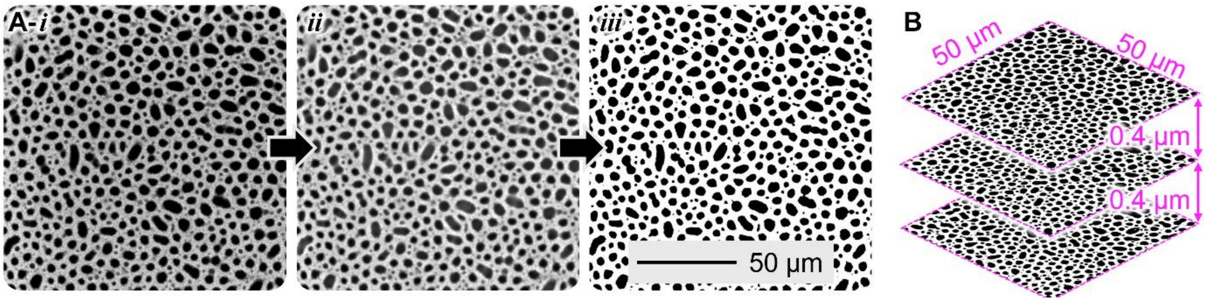

Figure S5. A Processing of CLSM images ***i*** showing the poly(*t*-BA) fluorescence of the bijel films; ***ii*** CLSM image after adjusting brightness and contrast and running a bandpass filter; ***iii*** binarized image after thresholding, with poly(*t*-BA) domains in white and water in black. **B** Calculating the poly(*t*-BA) volume by multiplying the white area with the distance of 0.4 µm between each *z*-slice.

S6. Effect of nanoparticle concentration on bijel film structure

Figure S6-A presents 3D-CLSM images of bijel films fabricated using varying SNP concentrations in the precursor mixture. The concentration ratio of CTA^+ to SNPs is maintained at 1.2 mM/wt% (equivalent to a total of 41 mM CTA^+ for 33 wt% SNPs). At decreasing SNP concentrations, the bijel films feature larger pores stretching from the top to the bottom surface. The CLSM micrographs in **Figure S6-B** acquired at mid-depth inside the films, reveal the coarsening of pores within the film structure.

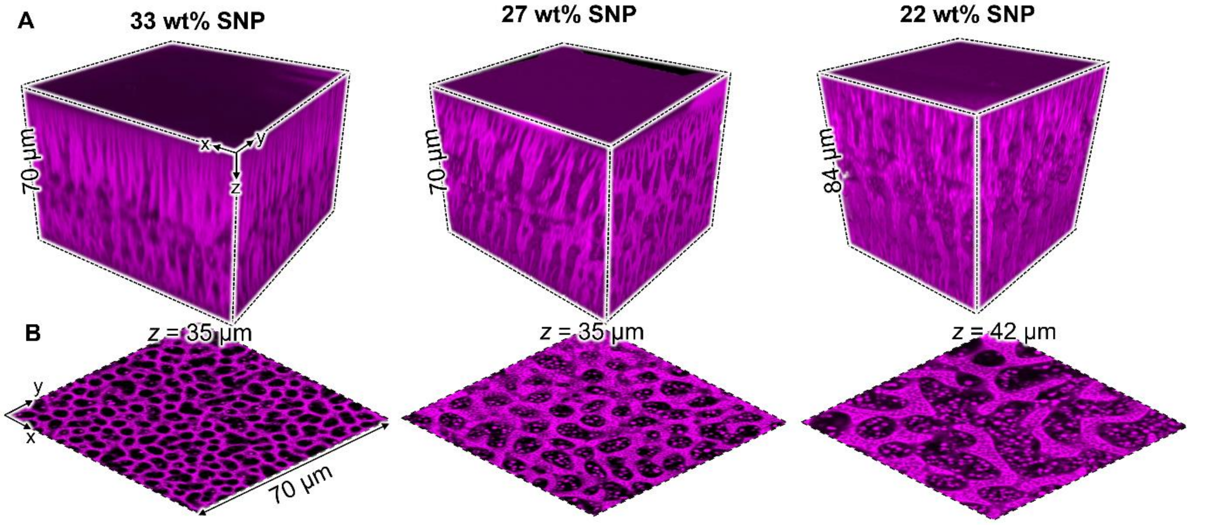

Figure S6. A 3D-CLSM images of bijel films prepared at different SNP concentrations in the precursor mixture. **B** CLSM slices taken at mid-depth within the film. Poly(*t*-BA-*co*-BDA) is colored in magenta and water in black.

S7. Silica nanoparticle hydrophobization by CTA^+

To probe the effect of increasing CTA^+ concentration on SNP hydrophobization, we determine the contact angle of *t*-BA with 8 wt% BDA on a SNP-coated surface in aqueous CTA^+ solutions. The contact angle provides a quantitative measure for the surfactant-induced hydrophobicity of the SNPs.

Figure S7-A shows the contact angle measurement setup. As SNP substrate, we spin-coat a 10 wt% SNP dispersion onto pre-cleaned microscopy slides according to the protocol in reference [1]. 350 µL of the Ludox® TMA dispersion is pipetted onto glass slides (Epredia) using a home-built spin-coater at

1000 rpm, followed by drying at 60 °C in an oven overnight. This spin-coating procedure produces dense and uniform SNP layers as demonstrated by SEM analysis in [1]. To remove potentially surface-active components from the *t*-BA/BDA solution, the acrylate mixture is purified three times using aluminum oxide powder (Honeywell Fluka™) and syringe filtration (0.2 µm, Minisart®; Sartorius). Due to the lower density of the *t*-BA/BDA solution compared to water, the acrylate is dispensed from below the SNP-coated microscopy slides into the aqueous bath containing 0.05 – 2 mM CTA^+ at pH 7. All contact angles are measured at neutral pH to probe CTA^+ concentrations below and above the CMC in water (~0.9 mM at pH 7), because the high ionic strength at acidic pH decreases the CMC for CTA^+ [2], [3]. The slides are immersed in the CTA^+ solution for 5 min prior to droplet deposition and contact angle measurement.

Droplets of 8 µL *t*-BA/BDA are dispensed onto the SNP-coated slides as presented in **Figure S8-B**. The contact angle is determined from side view imaging and fitting of the contact line at the interface of droplet, CTA^+ solution, and SNP substrate (SCA 20 software, Dataphysics OCA25). **Figure S8-C** shows increasing contact angles with increasing CTA^+ concentration, indicating that higher concentrations of CTA^+ can facilitate stronger SNP hydrophobization. As negative control, the contact angle of *t*-BA/BDA on the uncoated microscopy slide amounts to 39 ± 2°.

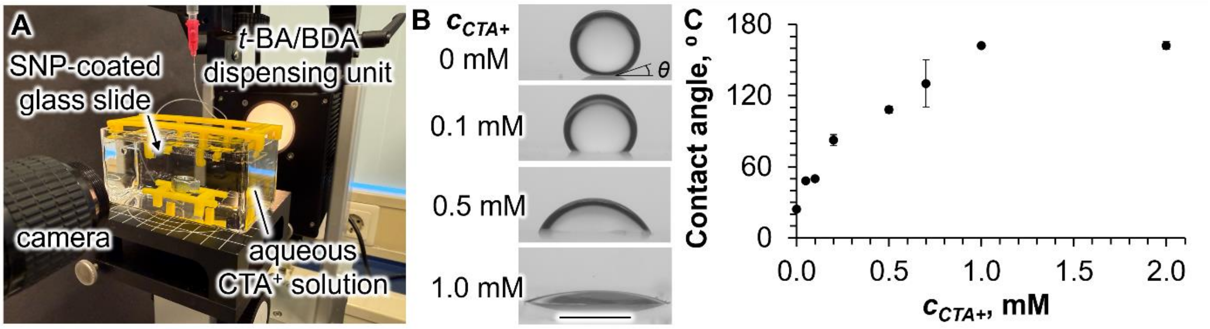

Figure S7. A Photograph of the contact angle measurement setup. **B** Brightfield images of *t*-BA/BDA droplets in aqueous CTA^+ solutions deposited onto SNP-coated microscopy slides. Black scale bar is 1 mm. **C** Average contact angles for *t*-BDA/BDA on SNP substrate for different CTA^+ concentrations. Error bars give the standard deviation calculated from the measurement of three droplets.

S8. Hydrogel swelling

CLSM analysis shows that the hydrogel structure remains similar to that of the poly(*t*-BA)-bijel before hydrolysis (**Figure S8**). Although the difference in refractive indices of polyacrylic acid (*n* = 1.44), SNPs (*n* = 1.46) and water (*n* = 1.33) along with the photobleaching of Coumarin 6 dye used for CLSM compromise the imaging quality, we conclude that hydrolysis does not degrade the bijel structure.

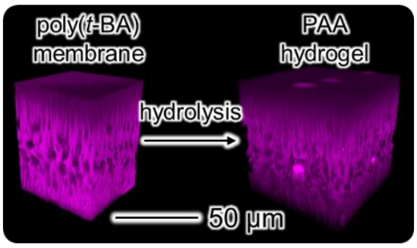

Figure S8. 3D-CLSM images of poly(*t*-BA-*co*-BDA) bijel film ($m_{BDA}/m_{t\text{-}BA}$ = 0.08; 41 mM CTA^+; imaged in DMSO) and PAA hydrogel (imaged in water pH 4) after hydrolysis. Poly(*t*-BA-*co*-BDA) and PAA are labelled in magenta and water in black.

We characterize the hydrogel swelling in water by measuring the area expansion of the hydrogel. To this end, the hydrogels are stored in water at pH 4 and cut into rectangular pieces using a razor blade (Reinforced razor blade No 44.50; Martor KG). Their width and length is measured with a caliper (Mitutoyo). The swelling process is initiated by the addition of 0.1 M NaOH to raise the pH of the water to pH 6 – 12. We determine the hydrogel area increase a from comparing the hydrogel width w_0 and length l_0 at pH 4 to the respective dimensions at pH 6 – 12 (denoted as w and l) *via* Equation 2:

$$a = \frac{(w*l)-(w_0*l_0)}{w_0*l_0} * 100\ \% \qquad \text{[Eq. 2]}$$

Figure S9 presents photographs of the hydrogel membrane ($m_{BDA}/m_{t\text{-}BA}$ = 0.08) during three cycles of swelling (pH 12) and deswelling (pH 4). Upon lowering the pH from 12 to 4, the hydrogel repeatedly shrinks in size and begins to fold. Although the swelling/deswelling behavior is reversible across the different cycles, the deformed hydrogels consistently rupture during flux tests.

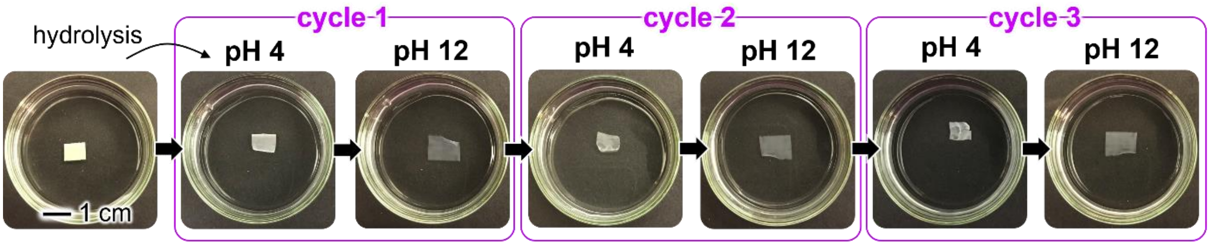

Figure S9. Photographs of the hydrogel membrane ($m_{BDA}/m_{t\text{-}BA}$ = 0.08) across three cycles of pH-induced swelling and deswelling.

S9. Microfiltration and water permeability measurements

The experimental setup for microfiltration and permeability testing is depicted in **Figure S10A**. To perform the filtration measurements, we craft a filtration device from 3D-printed parts. Our device enables leakage-free testing of the water flux of bijel and hydrogel membranes, as demonstrated in **Figure S10B** where red Acid Fuchsin solution is recovered solely in the water flow channel after filtration through a Nylon cloth. The filtration device which is built from a 3D-printed housing (designed with Autodesk Inventor 2021; printed with Formlabs 3BL printer using Clear Resin V4). **Figure S10C** shows the assembly of the device. For feed supply and permeate collection, we glue a needle (19 GA, KDS1912P; Weller) into the top and bottom parts using Clear resin V4 (UV-cured for 60 min) and seal them with layer of epoxy resin (Liqui Moly Epoxy Adhesive). The membrane sample is placed on a ~1 x 1 cm piece of Nylon microfiltration support (5 µm pores, 0.085-0.14 mm thick, RS10539; Tisch Scientific) to prevent mechanical rupture of the membrane during filtration. A 5 mm o-ring (2.5 mm thick, Duratool) at the top and a 19 mm o-ring (2.5 mm thick, Duratool) at the bottom part of the filtration housing prevent water leakages. The filtration assembly is fastened with four M4 screws, nuts, and

washers (Kelfort). We keep the hydrogel membranes wet during the entire assembly process.

The water permeability J (L/(h*m^2*bar)) is calculated from the mass of water $m(H_2O)$ (g) permeated through the poly(*t*-BA-*co*-BDA)/hydrogel membrane using Equation 3.

$$J = \frac{m(H_2O)}{\rho(H_2O)*t*A*p} \quad \text{[Eq. 3]}$$

With $\rho(H_2O)$ the density of water (0.99 g/mL), t the permeate collection time (2 – 5 min), A the membrane filtration area (0.2 cm^2) and the pressure of the water feed (bar).

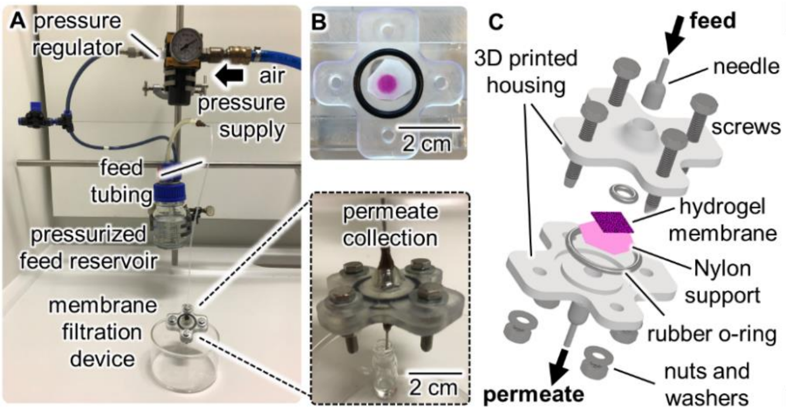

Figure S10. A Experimental setup for measuring the water permeability with close-up photo of the filtration device. **B** Photograph of the Nylon support after filtration with 0.02 g/L Acid Fuchsin solution. **C** Computer drawing of the filtration device.

To illustrate the SNP crust covering the poly(*t*-BA-*co*-BDA) membrane, **Figure S11** presents magnified SEM images of the membrane surface and cross-section. Dense SNP packings can be observed both at the membrane surface, with particles also deposited inside surface pores.

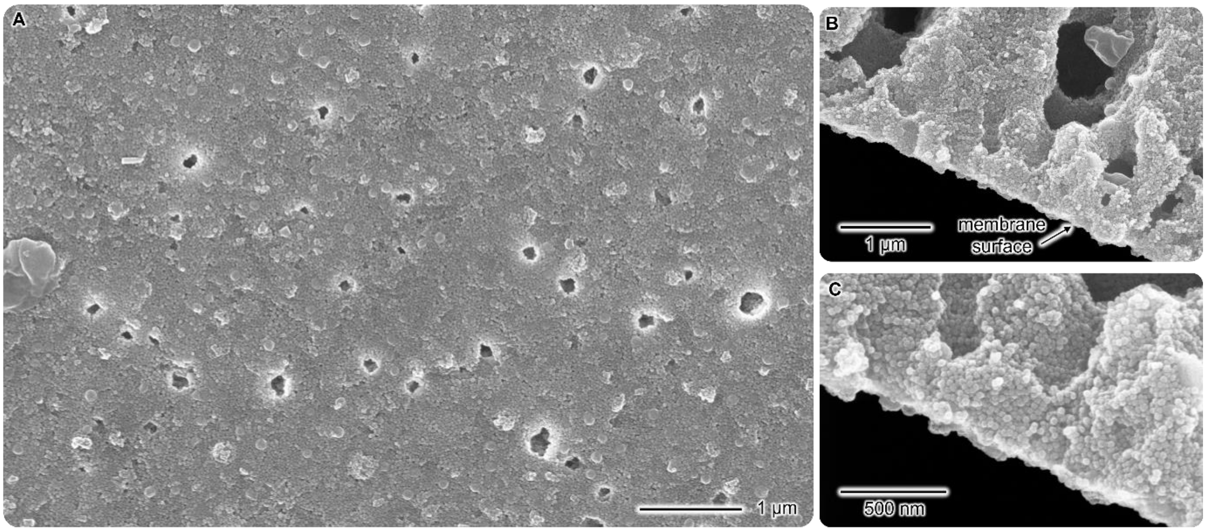

Figure S11. SEM images of **A** poly(*t*-BA-*co*-BDA) bijel membrane surface and **B** membrane cross-section at **C** higher magnification.

Figure S12 provides SEM micrographs of the poly(*t*-BA-*co*-BDA) bijel membrane before and after water filtration at a pressure of 4 bar which used in the microfiltration experiment. The membrane replicates retain structures comparable to those observed prior to filtration (for reference see also **Figure S4**). The preserved morphology suggests that the polymerized bijel membrane maintains its structure

under filtration pressures up to 4 bar.

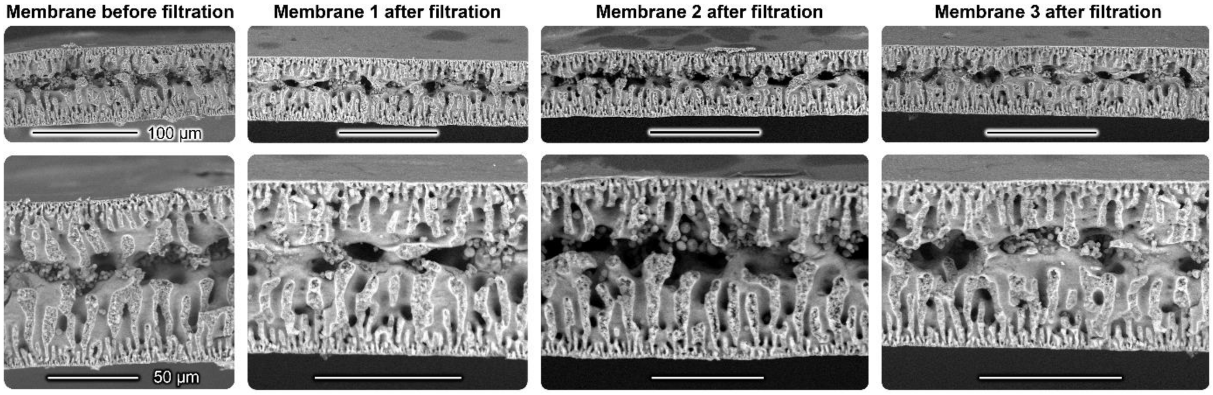

Figure S12. SEM images of the poly(*t*-BA-*co*-BDA) bijel before and the membrane after filtration at 4 bar. Black scale bars are 100 µm and white scale bars of the magnified insets below are 50 µm.

Figure S13A shows that the Nylon microfiltration support has a water permeability of 313 L/(min*cm^2) (> 240 L/(min*cm^2) reported by the manufacturer). The permeability of the Nylon support is significantly higher than the water permeability of the bijel membranes and bijel-derived hydrogels in **Figure 4D-F** in the manuscript. The Nylon support contains large pores as evident in the SEM images in **Figure S13B**.

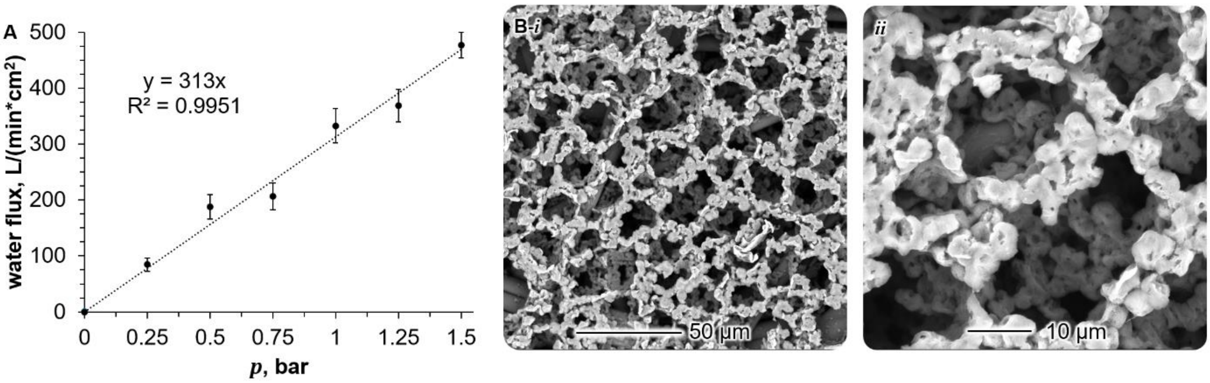

Figure S13. A Flux-pressure curve for Nylon microfiltration support. The slope of the linear fit gives the water permeability with R^2 the quality of the linear fit. The flux measurements are based on three membrane samples. **B-*i*** SEM image of the Nylon microfiltration support and ***ii*** magnified inset.

S10. Hydrogel surface porosity before and after filtration

CLSM analysis is used to image the hydrogel surface pores (acquired at $z \approx 5$ µm below the top surface) before and after flux testing at a pressure of 1 bar. To this end, the fluorescence dye Nile red is replaced by Coumarin 6 in bijel synthesis. The confocal micrographs in **Figure S14** feature black water pores surrounded by magenta PAA for all cross-linker ratios $m_{BDA}/m_{t\text{-}BA}$. The pore morphologies are similar before and after filtration.

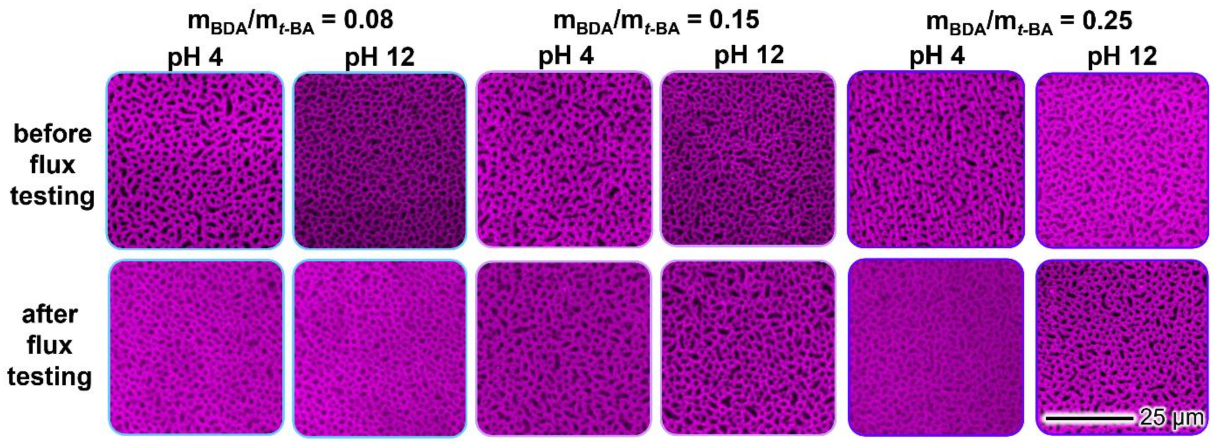

Figure S14. CLSM images of the hydrogel pores at $z \approx 5$ µm below the top surface before and after flux testing at 1 bar.

S11. Characterization of filtration feed dispersions

To synthesize the SNP/polymer composite particles, 9.93 g BDA is mixed with 0.53 g hexadecane (99 % pure; Thermo Fisher) and 0.05 g 2,2-azobisisobutyronitrile (Sigma Aldrich) and labelled with the fluorescence dye Coumarin 6. The liquid mixture is added to 80 mL of 20 wt% Ludox® TMA dispersion (34 wt% colloidal silica, 20 nm; Sigma Aldrich) containing 0.5 mM CTA^+. Emulsification is achieved using a tip-sonicator operated at 50 % amplitude (250 W; 10 mm tip diameter; Qsonica LCC) for 2 min. The Ludox® TMA-stabilized BDA droplets are polymerized by heating to 70 ºC in a polyethylene glycol bath (PEG400; Sigma Aldrich) overnight.

The polymerized composite particles are washed to remove CTA^+ and residual Ludox® TMA particles by centrifugation at 5000 rcf for 15 min (Avanti™ J-20 XP; Beckman Coulter). The supernatant is discarded, and the sediment is re-dispersed in 15 mL water adjusted to pH 2 (MilliQ purification) using a spatula and vortexer. The redispersed emulsion is centrifuged at 10000 rcf for 20 min and the supernatant is discarded. Redispersing the concentrated sediment produces a SNP/polymer composite particle dispersion with a weight fraction 45 wt%. For filtration experiments, the composite particle stock is diluted in water (pH 4) to obtain a 2 wt% dispersion. Additionally, a 5 wt% Ludox® TMA (34 wt% stock; Grace, batch 1000374513) dispersion is prepared by dilution in water of pH 4.

Dynamic light scattering is employed to characterize the size distribution of the SNP/polymer composite and Ludox® TMA particles. **Figure S15A** and **B** shows that the mean particle sizes are 251 ± 4 nm for the composite particles (polydispersity index (PDI) 12 ± 3 %) and 9 ± 1 nm for the Ludox TMA® particles (PDI 21 ± 1 %). The measured Zeta-potential of the SNP/polymer composite particles (pH 4) is -32 ± 2 mV, which is comparable to that of the Ludox® TMA particles (pH 4) of -34 ± 1 mV.

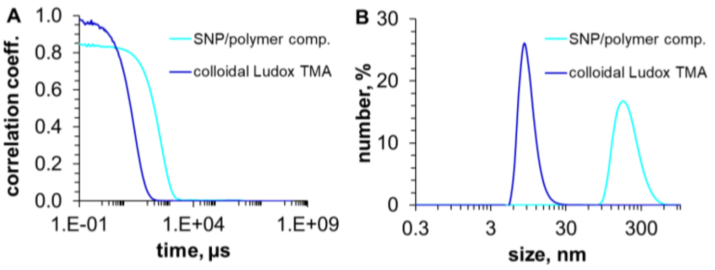

Figure S15. A Correlation coefficient and **B** size distribution of SNP/polymer composite and Ludox® TMA particles obtained from dynamic light scattering.

Table S3 summarizes the concentrations of SNP/polymer composite and Ludox® TMA particles in feed, blank, and permeate samples of the microfiltration experiment.

Table S3. Particle concentrations in feed, blank, and permeate samples during microfiltration.

	**concentration of SNP/polymer composite particles**	**concentration of Ludox® TMA**
**Feed**	2.0 wt%	5.2 wt%
**Blank**	2.0 wt%	5.2 wt%
**Permeate 1**	0.1 wt%	4.7 wt%
**Permeate 2**	0.1 wt%	4.3 wt%
**Permeate 3**	0.1 wt%	4.8 wt%

References

[1] H. Siegel, M. de Ruiter, T. H. R. Niepa, M. F. Haase. The effect of charge screening for cationic surfactants on the rigidity of interfacial nanoparticle assemblies. *J. Colloid Interface Sci.* 2025, 678, 201–208

[2] M. J. Qazi, S. J. Schlegel, E. H. G. Backus, M. Bonn, D. Bonn, N. Shahidzadeh. Dynamic Surface Tension of Surfactants in the Presence of High Salt Concentrations. *Langmuir* 2020, 36, 27, 7956–7964

[3] J. M. Goronja, A. M. Janošević Ležaić, B. M. Dimitrijević, A. M. Malenović, D. R. Stanisavljev, N. D. Pejić. Determination of critical micelle concentration of cetyltrimethyl-ammonium bromide: Different procedures for analysis of experimental data. *Hem. Ind*. 2016, 70, 4, 485–492
